# Chiavari Letter

**Published:** 1887-08

**Authors:** A. Lagorio

**Affiliations:** Chiavari, Italy


*Mr. Editor of the Chicago Medical Journal and Examiner:*

THE obsterical clinique at Pammatone Hospital in Genoa has just closed, and a few notes taken from it will not fail, I hope, to be of interest to your readers. The clinique was opened on Nov. 11th, 1886, and was closed on June 24th, 1887. There have been admitted 118 women; of these 106 gave birth to children, five left the hospital before going through labor, six remained in the hospital at the time of its closure, and one showed symptoms of abortion. Many pregnancies were complicated with pulmonary tuberculosis, syphilis, enormous œdema of the lower extremities, epilepsy, varicose swellings, bronchitis, mania, and hæmorrhoids. Three gave birth to twins; 60 were unmarried, 16 widows, 52 primiparæ, 66 multiparæ, and some were public prostitutes.

The vertex presentations were 83, facial, 3, and pelvic, 7. Premature births were six, and one abortion. Professor Pajot's forceps were used four times in cranial presentations; once only was version resorted to for a trunk presentation, in a twin pregnancy.

In one of the three named twin gestations there was found a male and a female with double presentation of the vertex; in another, a male and a female with a presentation of the cranium and with a presentation of the nates; in the third, two females, one of whom presented with the hips, and the other with the right shoulder and procidentia of the corresponding hand.

The amniotic sac, because of its resistance, had to be broken, artificially, four times. The shortest umbilical cord was 27 centimeters, the longest 103 centimeters; the cord was once knotted, twice prolapsed, and 28 times coiled around the neck of the child.

Of the children, one had hydrocephalus, and another was affected with anencephalia and spina bifida in the cervical regions.

Many labors were complicated with metrorrhagia due to uterine inertia, treated with ergot, quinine, electricity, wine, and Credé's method.

The puerperium was also complicated with eclampsia, peritonitis, mania, metrorrhagia, agalaxia, mastitis, fissured nipples, etc.

Two deaths occurred in 118 patients; one during labor, bloodless, and anæmic and without being liberated; the other by typhoid fever, fourteen days after a compound and artificial labor.

Of the new-born, some showed slight jaundice, and quite often many were affected with purulent ophthalmia, due to cold and insufficient nutrition; Neisser's gonococcus was not found. Five died of inanition, although nursed in suitable cradles according to the Parisian method.

On page 513 of the second volume of the "Practical Treatise on Obstetrics," by A. Charpentier, we read: "When, during pregnancy, one is convinced that a trunk presentation exists, cephalic version must be performed and maintained by all possible means." This practice and teaching Professor Macari does not approve. He criticises and condemns it. In a case of trunk presentation, the professor followed his own plan of *letting alone and waiting*.

On the 3d of last January a woman 33 years old, married, multipara, of a lymphatic and highly nervous constitution, was admitted into the obstetrical ward. She was in the seventh month of her sixth gestation; her first four pregnancies ended in premature, spontaneous labors, the fifth terminated normally. An irregular presentation of the fœtus was suspected. An accurate examination was made by inspection, palptation, percussion, abdominal ausculation, vaginal examination and pelvimetry. It was found that the patient had a simple uterine pregnancy, with living fœtus; oblique presentation, right shoulder in the second position, with the head to the right, lower than the pelvic extremity of the fœtus. The maternal pelvis was regularly developed. The professor concluded to wait, and abstain from any operative act.

After some time the same diagnosis was confirmed, and it was again decided *to wait.*

On the 9th of March labor began. By vaginal examination there was found a cranial presentation. At 9:45 P. M. of the same day the patient gave birth to a mature fœtus, male, sound and healthy, 56 centimeters long, 3,900 grams in weight; vertex presentation in second position; length of labor, 30 hours.

Professor Francisco Macari holds the Chair of Obstetrics and Gynæcology in the University of Genoa, and is director of the obstetrical clinique. His lectures are largely attended by students and midwives, for they are practical full of common sense. His work on obstetrics, gynæcology and pediatrics is concise and well written. It has been translated into French and German, and has been honored with three editions. Professor Macari doesn't think much of germs. During the cholera epidemic of Genoa, two years ago, he offered to swallow a certain amount of comma-bacilli to prove their innocuosness.

Professors M. Semmola of Naples, and F. Durante of Rome, will be the Italian delegates to the International Medical Congress at Washington next September. Professor Semmola is preparing an important paper on bacteriology. Professor Durante will protract his visit two months, having been ordered by his government to make a scientific tour through the United States; to visit hospitals and medical institutions, and to make a report. The Professor holds the Chair of Surgery at Rome, and enjoys a widespread and well-deserved reputation for his bold and skillful operations. He is a fine looking man and a fluent speaker. Chicago, always foremost and so well known for noble enterprises and generous hospitality, will not fail, I hope, to entertain and render due honors, to the brilliant surgeon of our Eternal City.

A. LAGORIO.

CHIAVARI, ITALY, July 10th, 1887.

